# Supplementary material for: Clinical prediction models for the management of blunt chest trauma in the emergency department: a systematic review
Source: BMC Emerg Med. 2024 Oct 12;24:189. doi: 10.1186/s12873-024-01107-6 (PMC11470733; doi:10.1186/s12873-024-01107-6)
Supplement: Supplementary file 1 — Supplementary Material 1 [file 12873_2024_1107_MOESM1_ESM.docx]

Example search strategy using PubMed from 1^st^ Jan 2000 until 1^st^ April 2024.

Medical Subject Heading terms, text words and word variants for blunt chest trauma

**MESH terms**

| **Keyword search in MeSH** | **MeSH term (s) found** |
| --- | --- |
| Blunt chest trauma | No items found |
| Blunt thoracic trauma | No items found |
| non-penetrating chest trauma | No items found |
| non-penetrating thoracic trauma | No items found |
| Blunt | Wounds, nonpenetrating |
| Chest | Thorax  Thoracic injuries  Thoracic wall |
| Trauma | Wounds and injuries |
| Trauma management | No items found |
| Risk prediction | No items found |
| Risk model | No items found |
| Risk prediction | No items found |
| Chest injury | Thoracic injury |
| Blunt chest injury | No items found |
| Stratification | No items found |
| ROC curve | ROC curve |
| Discriminat* | ROC curve |
| C statistic | No items found |
| AUC | Area under curve |
| Calibration | Calibration |
| Indices | Trauma Severity Indices |
| Algorithm | Algorithms |
| Multivariable | No items found |
| Variate | Canonical Correlation Analysis |
| Predict* | Clinical Decision Rules |
| Rule* | Clinical Decision Rules |
| Outcome* | Patient Outcome Assessment  Critical Care Outcomes |
| Risk* | Risk  Risk Factors |
| Model* | Models, statistical |
| Mortality | Mortality |
| Survival | Survival |
| Complications | "complications" [Subheading] |

| **Pubmed search from 1^st^ Jan 2000 until 1^st^ April 2024** |
| --- |
| "Wounds, Nonpenetrating"[Mesh] |
| "Thoracic Injuries"[Mesh] |
| "Wounds, Nonpenetrating"[Mesh] AND "Thoracic Injuries"[Mesh] |
| "ROC Curve"[Mesh] |
| "area under Curve"[Mesh] |
| "calibration"[Mesh] |
| "Trauma Severity Indices"[Mesh] |
| "algorithms"[Mesh] |
| "Canonical Correlation Analysis"[Mesh] |
| "Clinical Decision Rules"[Mesh] |
| "Patient Outcome Assessment"[Mesh] |
| "Critical Care Outcomes"[Mesh] |
| "risk"[Mesh] |
| "Risk Factors"[Mesh] |
| "Models, statistical"[Mesh] |
| "Mortality"[Mesh] |
| "survival"[Mesh] |
| "complications" [Subheading] |
| “animals” [Mesh] |
| “child” [Mesh] |
| "Wounds, Nonpenetrating"[Mesh] AND "Thoracic Injuries"[Mesh] AND "ROC Curve"[Mesh] |
| "Wounds, Nonpenetrating"[Mesh] AND "Thoracic Injuries"[Mesh] AND "area under Curve"[Mesh] |
| "Wounds, Nonpenetrating"[Mesh] AND "Thoracic Injuries"[Mesh] AND "calibration"[Mesh] |
| "Wounds, Nonpenetrating"[Mesh] AND "Thoracic Injuries"[Mesh] AND "Trauma Severity Indices"[Mesh] |
| "Wounds, Nonpenetrating"[Mesh] AND "Thoracic Injuries"[Mesh] AND "algorithms"[Mesh] |
| "Wounds, Nonpenetrating"[Mesh] AND "Thoracic Injuries"[Mesh] AND "Canonical Correlation Analysis"[Mesh] |
| "Wounds, Nonpenetrating"[Mesh] AND "Thoracic Injuries"[Mesh] AND "Clinical Decision Rules"[Mesh] |
| "Wounds, Nonpenetrating"[Mesh] AND "Thoracic Injuries"[Mesh] AND "Patient Outcome Assessment"[Mesh] |
| "Wounds, Nonpenetrating"[Mesh] AND "Thoracic Injuries"[Mesh] AND "Critical Care Outcomes"[Mesh] |
| “Wounds, Nonpenetrating"[Mesh] AND "Thoracic Injuries"[Mesh] AND "risk"[Mesh] |
| "Wounds, Nonpenetrating"[Mesh] AND "Thoracic Injuries"[Mesh] AND "risk factors"[Mesh] |
| "Wounds, Nonpenetrating"[Mesh] AND "Thoracic Injuries"[Mesh] AND "Models, statistical"[Mesh] |
| "Wounds, Nonpenetrating"[Mesh] AND "Thoracic Injuries"[Mesh] AND "Mortality"[Mesh] |
| "Wounds, Nonpenetrating"[Mesh] AND "Thoracic Injuries"[Mesh] AND "Survival"[Mesh] |
| "Wounds, Nonpenetrating"[Mesh] AND "Thoracic Injuries"[Mesh] AND "complications" [Subheading] |
| "Wounds, Nonpenetrating"[Mesh] AND "Thoracic Injuries"[Mesh] AND "ROC Curve"[Mesh]) **OR "area under Curve"[Mesh] OR "Trauma Severity Indices"[Mesh]) OR "algorithms"[Mesh]) OR "Clinical Decision Rules"[Mesh]) OR "Patient Outcome Assessment"[Mesh]) OR "risk"[Mesh]) OR "risk factors"[Mesh]) OR "Models, statistical"[Mesh]) OR "Mortality"[Mesh]) OR "complications" [Subheading])** |
| "Wounds, Nonpenetrating"[Mesh] AND "Thoracic Injuries"[Mesh] AND "ROC Curve"[Mesh] OR "area under Curve"[Mesh] OR "Trauma Severity Indices"[Mesh] OR "algorithms"[Mesh]) OR "Clinical Decision Rules"[Mesh] OR "Patient Outcome Assessment"[Mesh] OR "risk"[Mesh] OR "risk factors"[Mesh] OR "Models, statistical"[Mesh] OR "Mortality"[Mesh] OR "complications" [Subheading] **NOT “animals”[Mesh]** |
| "Wounds, Nonpenetrating"[Mesh] AND "Thoracic Injuries"[Mesh] AND "ROC Curve"[Mesh] OR "area under Curve"[Mesh] OR "Trauma Severity Indices"[Mesh] OR "algorithms"[Mesh]) OR "Clinical Decision Rules"[Mesh] OR "Patient Outcome Assessment"[Mesh] OR "risk"[Mesh] OR "risk factors"[Mesh] OR "Models, statistical"[Mesh] OR "Mortality"[Mesh] OR "complications" [Subheading] NOT “animals”[Mesh] **NOT “child”[Mesh]** |
| "Wounds, Nonpenetrating"[Mesh] AND "Thoracic Injuries"[Mesh] NOT "animals"[Mesh] NOT "child"[Mesh] |
